# Delayed diagnosis of Muckle-Wells syndrome – analysis of influencing factors

**DOI:** 10.1186/1546-0096-10-S1-A88

**Published:** 2012-07-13

**Authors:** Jasmin B Kuemmerle-Deschner, Samuel D Samba, Isabelle Koné-Paut, Isabelle Marie, Katharina Gramlich, Sandra Hansmann, Theodoros Xenitidis, Susanne M Benseler

**Affiliations:** 1 Bicêtre University Hospital, le Kremlin Bicetre, lle-de-France, France; 2 The Hospital for Sick Children, Toronto, ON, Canada; 3 University Hospital Tubingen, Tuebingen, BW, Germany

## Purpose

Muckle-Wells syndrome (MWS) is a rare inherited autoinflammatory disease. Patients may present with fever, rash, arthralgia, or conjunctivitis. Effective treatment of MWS has become available with the advance of IL-1 inhibition preventing disease sequalae of sensorineural deafness and amyloidosis. Therefore, early diagnosis of MWS is crucial to prevent -organ damage. The aim of this study was to identify key factors contributing to a delay in diagnosis of MWS.

## Methods

In two rheumatology centers a cohort of consecutive children and adults with genetically confirmed MWS were interviewed using a previously developed standardized questionnaire. The tool captures a total of 55 variables including patient related demographic factors, referral process related variables and presenting MWS symptoms at time of MWS diagnosis.

## Results

A total of 32 patients, 18 females/14 males were included. These were 10 children and 22 adults with active MWS and confirmed mutations of the NLRP3 gene. The median age was 36 years (range 3-75). The median distance from home to the rheumatology center was 20km (range 7-577). The mean time elapsed between first consultation and final diagnosis was 21.9 years (range 0 – 63 years). The major symptoms reported by the patients were musculoskeletal (75%), skin disease (63%), eye disease (47%), relapsing fevers (41%) and hearing loss (34%). Diagnoses preceding the correct recognition of MWS included rheumatic disease (41%), conjunctivitis (41%), hearing loss (31%) and urticaria (28%). No definite diagnosis was made in 25%. Medical subspecialties most frequently consulted first were pediatricians (38%) and general practitioners (31%). In 84% the physician to establish the diagnosis of MWS was a rheumatologist.

## Conclusion

In patients with MWS, diagnosis is dramatically delayed most likely due to low level of knowledge regarding the disease. As severe disease sequelae like sensorineural deafness and amyloidosis may be prevented by early diagnosis and effective IL-1 inhibition the education of medical professionals in the area of autoinflammatory diseases needs to be intensified. Designated reference centers may contribute in accelerating the process of diagnosis and therapy.

## Disclosure

Jasmin B. Kuemmerle-Deschner: None; Samuel D. Samba: None; Isabelle Koné-Paut: None; Isabelle Marie: None; Katharina Gramlich: None; Sandra Hansmann: None; Theodoros Xenitidis: None; Susanne M. Benseler: None.

